# Laser Speckle Contrast Analysis: Functional Evaluation of Microvascular Damage in Connective Tissue Diseases. Is There Evidence of Correlations With Organ Involvement, Such as Pulmonary Damage?

**DOI:** 10.3389/fphys.2021.710298

**Published:** 2021-10-11

**Authors:** Barbara Ruaro, Cosimo Bruni, Barbara Wade, Elisa Baratella, Paola Confalonieri, Caterina Antonaglia, Pietro Geri, Marco Biolo, Marco Confalonieri, Francesco Salton

**Affiliations:** ^1^Unit of Pulmonology, University Hospital of Trieste, Trieste, Italy; ^2^Department of Experimental and Clinical Medicine, Division of Rheumatology, Careggi University Hospital, University of Florence, Florence, Italy; ^3^AOU City of Health and Science of Turin, Department of Science of Public Health and Pediatrics, University of Turin, Turin, Italy; ^4^Department of Radiology, Department of Medicine, Surgery and Health Science, University of Trieste, Trieste, Italy

**Keywords:** systemic sclerosis, systemic lupus erythematosus, peripheral microcirculation, laser speckle techniques, blood perfusion, digital ulcers

## Abstract

Laser speckle contrast analysis (LASCA) is a non-contact technique able to quantify peripheral blood perfusion (PBP) over large skin areas. LASCA has been used to study hand PBP in several clinical conditions. These include systemic sclerosis (SSc) and systemic lupus erythematosus (SLE) and LASCA showed that PBP was significantly lower in these conditions than in healthy subjects (HS). Moreover, it has been demonstrated that LASCA is a safe technique also able to monitor digital ulcer perfusion and their evolution in SSc patients, during systemic and local treatment. The use of LASCA, coupled with reactivity tests is commonplace in the field of microvascular function research. Post-occlusive hyperemia reactivity (POHR) and local thermal hyperemia, associated with laser techniques are reliable tests in the evaluation of perfusion in SSc patients. Other studies used laser speckled techniques, together with acetylcholine and sodium nitroprusside iontophoresis, as specific tests of endothelium function. In conclusion, LASCA is a safe, non-contact reliable instrument for the quantification of PBP at skin level and can also be associated with reactivity tests to monitor disease progression and response to treatment in different connective tissue diseases.

## Highlights

– Vasculopathy is a hallmark of several connective tissue diseases, such as systemic sclerosis (SSc) and systemic lupus erythematosus (SLE).
– Laser speckle contrast analysis (LASCA) is a non-contact, safe technique able to quantify peripheral blood perfusion at different skin areas in SSc and SLE patients.
– LASCA, alone or together with reactivity tests, is useful for the monitoring of disease progression, response to treatment and ulcer outcome.
– LASCA is able to predict major vascular complication such as digital ulcers (DU).
– LASCA can help to follow DU treatment and outcome.

## Introduction

Microvascular dysfunction is a key component in several connective tissue diseases, including systemic sclerosis (SSc) and systemic lupus erythematosus (SLE) and has important clinical implications, e.g., Raynaud’s Phenomenon (RP) (Lin et al., 2004; Minier et al., 2014; Sebastiani et al., 2014; Bruni et al., 2018; Cutolo et al., 2018; Smith et al., 2018; Tamirou et al., 2018; Meroni and Tsokos, 2019; Saygin et al., 2019). Indeed, a morphological and functional assessment of the peripheral microvasculature in SSc and SLE patients is a diagnostic necessity, as it also plays a prognostic and therapeutic role (Wigley et al., 1990; Roustit and Cracowski, 2012; Sulli et al., 2014a; Wigley and Flavahan, 2016; Ruaro et al., 2018a; Shirazi et al., 2021). Nailfold videocapillaroscopy (NVC) is a validated method to assess peripheral microangiopathy at the nailfold level, whilst laser speckle contrast analysis (LASCA) allows for a functional evaluation of peripheral microcirculation over large skin areas (Wigley et al., 1990; Roustit and Cracowski, 2012; Sulli et al., 2014a; Wigley and Flavahan, 2016; Ruaro et al., 2018a; Shirazi et al., 2021). LASCA quantifies the peripheral blood perfusion (PBP) in perfusion units (PU) and is a non-contact, rapid, reproducible and high-resolution technique (Ruaro et al., 2014, 2015, 2016, 2019a; Barsotti et al., 2020; Hughes et al., 2020; Shirazi et al., 2021).

Early biomedical application of laser speckle techniques dates back to 1981. This was followed by continuous research and enhancement in the field of laser speckle analysis (Asakura and Takai, 1981).

**FIGURE 1:**
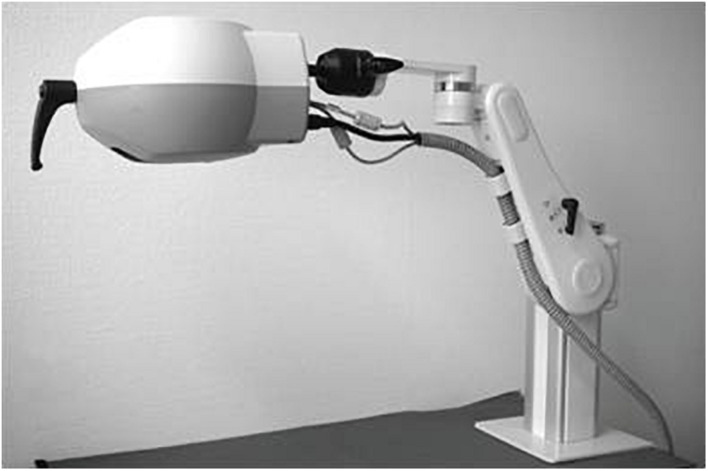
Laser speckle contrast analysis (LASCA) is composed of: a head, an adjustable arm, a detector camera and a software for data acquisition and analysis. (Courtesy by Perimed).

Laser speckle contrast analysis (Pericam PSI, Perimed, Jarfalla) (Figure 1) visualizes tissue microvascular blood perfusion instantaneously and is based on the principle that when an object is illuminated by laser light, the backscattered light forms a random interference pattern which has dark and bright areas, producing a so-called “speckle pattern.” This *speckle pattern* is stationary if a static object is illuminated, whilst if a moving object is illuminated, e.g., red blood cells inside human tissue, then the speckle pattern observed will fluctuate in intensity. This system is fitted with a CCD camera able to record these changes and quantify them into technical parameters, i.e., working distance, point density, frame rate resolution and provide the width and height of the measurement area.

**FIGURE 2:**
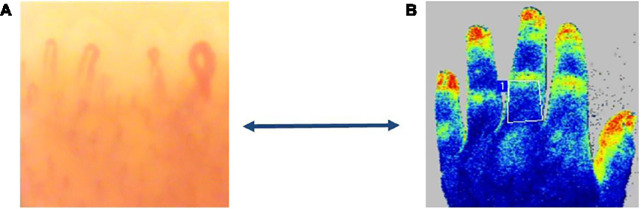
Microvascular damage in systemic sclerosis. Evaluation of peripheral blood perfusion (PBP) by Laser Speckled Contrast Analysis (LASCA) in a patients with an “Early” capillaroscopy pattern **(A)**. Regions of interest (white line) created for evaluation of blood perfusion (BP) by LASCA (blue color, low BP, yellow color, intermediate BP, red color, higher BP) in a SSc patient **(B)** (Courtesy by Perimed).

During and after the LASCA registration, it is also possible to create different regions of interest (ROI) and times of interest (TOI) to evaluate the perfusion (Ruaro et al., 2014, 2015, 2016, 2019a; Barsotti et al., 2020; Hughes et al., 2020; Figure 2).

Although laser speckle contrast imaging (LSCI) is similar to LASCA, the contrast is calculated on a single pixel over a number of time frames. Although it has a spatial resolution which is five-times bigger than that of LASCA, it has a poor temporal resolution. Moreover, as calculations are made over a number of frames, processing takes a bit longer than LASCA (Gaillard-Bigot et al., 2014; Sebastiani et al., 2014; Pauling et al., 2015; Vaz et al., 2016; Zötterman et al., 2017; Jeon et al., 2020).

This review aims at updating information on the new acquisition regarding the laser speckle techniques in the study of functional microvascular alterations in connective tissue diseases for the diagnosis, follow-up and management of treatment in these complex conditions. Moreover, it also assesses any correlations between skin perfusion and organ involvement, such as pulmonary damage.

## Laser Speckle Contrast Analysis in Systemic Sclerosis

### Correlation With Capillaroscopy

Systemic sclerosis is characterized by structural and functional vasculopathy, where the peripheral microvascular involvement may manifest as Raynaud’s phenomenon (RP) and/or digital ulcers (DU) (Domsic et al., 2014; Gaillard-Bigot et al., 2014; Sebastiani et al., 2014; Sulli et al., 2014b; Lambrecht et al., 2016; Morales-Cárdenas et al., 2016; Trombetta et al., 2016; Smith et al., 2018; Frech and Murtaugh, 2019; Bruni et al., 2020; Guigui et al., 2020; Farina et al., 2021; Gigante et al., 2021; Suliman et al., 2021). Frequently, RP is the earliest morphological and functional manifestation of SSc vasculopathy (Domsic et al., 2014; Gaillard-Bigot et al., 2014; Sulli et al., 2014b; Lambrecht et al., 2016; Morales-Cárdenas et al., 2016; Trombetta et al., 2016; Frech and Murtaugh, 2019; Guigui et al., 2020; Farina et al., 2021; Gigante et al., 2021; Suliman et al., 2021). RP, secondary to SSc (SRP), is also the most common presenting feature of the disease, it is observed in 95% of scleroderma patients (Wigley et al., 1990; Wigley and Flavahan, 2016; Bruni et al., 2018). During RP, the skin usually turns white (ischemia), blue (deoxygenation) and then red (reperfusion) (Wigley et al., 1990; Wigley and Flavahan, 2016). SRP occurs in response to cold temperature or emotional stress, in the setting of underlying vascular disturbance and is often associated with digital pain and ischemic ulcers (Wigley et al., 1990; Wigley and Flavahan, 2016; Bruni et al., 2018). It has been postulated that progressive deficiency in the vasodilatory capacity of the vessels, intimal fibroproliferation, thrombosis of the digital arteries and tissue ischemia may well be the underlying mechanisms that lead to persistent vascular involvement (Domsic et al., 2014; Wigley and Flavahan, 2016; Bruni et al., 2018; Ruaro et al., 2019a). Endothelial cell injury and activation lead to vascular dysfunction and vasospasm that may quickly obstruct the already limited blood flow of the vasculopathic digital arteries (Wigley et al., 1990; Roustit et al., 2010; Pauling et al., 2012; Della Rossa et al., 2013, 2016; Wigley and Flavahan, 2016; Bruni et al., 2018, 2020; Wilkinson et al., 2018; Ruaro et al., 2019b; Farina et al., 2021). There is often a luminal narrowing of > 75% of digital arteries due to underlying intimal fibrosis and luminal occlusion caused by thrombi (Wigley et al., 1990; Wigley and Flavahan, 2016; Bruni et al., 2018; Farina et al., 2021).

Furthermore, degradation of the endothelial barrier occurs in response to perivascular inflammation and to reactive oxygen species generated by the ischemia-reperfusion injury, taking place in SSc patients’ microvasculature. This phenomenon is a trigger to the endothelial barrier leading to opening of the endothelial junctions, inflammatory cells homing, sustained hyperpermeability and continuous vascular leak (Wigley et al., 1990; Wigley and Flavahan, 2016; Bruni et al., 2018; Farina et al., 2021).

Today, the validated method to study the morphological vascular alteration in SSc patients is NVC but several authors reported the utility of LASCA in the evaluation of functional damage of microvascular system (Ruaro et al., 2014, 2015, 2016, 2019a; Barsotti et al., 2020; Hughes et al., 2020; Shirazi et al., 2021).

Ruaro et al. (2014) evaluated 61 SSc patients and 61 healthy subjects (HS) by NVC, LASCA and single laser Doppler flowmetry (LDF) at the fingertip level. The peripheral blood perfusion (PBP), detected by LASCA and LDF, was lower in SSc patients than in HS (*p*-value < 0.0001), with a progressive decrease along the “Early,” “Active,” and “Late” NVC patterns. Although a positive correlation was observed between LASCA and LDF values in all subjects, LASCA has a lower intra-operator variability than LDF, is able to evaluate larger skin areas, is significantly less time consuming and more readily accepted by patients (Ruaro et al., 2014; Table 1).

**TABLE 1:** Milestone in the study of peripheral blood perfusion studied by LASCA in systemic sclerosis and systemic lupus erythematosus.

	First Author	Study population (number)	Summary of results
LASCA in systemic sclerosis	Ruaro et al., 2014	61 SSc; 61 HS	LASCA detected lower PBP at the level of fingertip in SSc patients than in healthy subjects and LASCA perfusion values were found correlated with progression of NVC patterns of microangiopathy
	Sulli et al., 2014b	70 SSc; 70 HS	Perfusion assessed by LASCA, at different areas of the face (forehead, tip of nose, zygomas and perioral region) and at dorsal and volar aspects of hands was significantly lower than in healthy subjects at fingertips, periungual areas and palm of hands (*p* < 0.0001).
	Ruaro et al., 2016	61 SSc; 61 HS	A gradual decrease of peripheral blood perfusion at fingertips, periungual and palm areas, was found in SSc patients with progressive severity of NVC patterns of microangiopathy (“Early,” “Active,” or “Late”) (*p* < 0.01). A negative correlation was observed between PBP values and loss of capillaries in the three reported areas of hands
	Ruaro et al., 2019a	31 PRP, 70 SSc and 68 HS	Both PRP and SSc showed a statistically significant lower PBP than HS at the level of fingertips, periungual, palmar aspect of 3rd finger and palm areas. BP was significantly lower in PRP than in SSc with the “Early” pattern of microangiopathy in the same areas above reported
	Farina et al., 2021 Della Rossa et al., 2013	20 primary RP 36 SSc and 20 HS	LASCA was assessed at the level of hand dorsum at baseline and after cold test and post-occlusive hyperemia test. The researchers reported that after cold test, SSc had a significant reduction of blood flow as compared to HS. Furthermore SSc patients presented a statistically significant higher recovery time compared to HS and PRP
	Della Rossa et al., 2016	60 SSc (10 VEDOSS and 50 full-blown SSc)	LASCA was assessed at the level of hand dorsum at baseline and after cold test and post-occlusive hyperemia test. The researchers reported that after cold test, SSc had a significant reduction of blood flow as compared to HS. Furthermore SSc patients presented a statistically significant higher recovery time compared to HS and PRP
	Jeon et al., 2020	10 SSc; 5 HS	Peri-oral and lip LSCI measurements were made in who had facial and peri-oral fibrosis limiting their mouth opening. However no significant difference was found between SSc and HS at peri-oral area
	Pauling et al., 2012; Pauling et al., 2015	25 SSc and 18 PRP	Digital perfusion was moderate-to-good correlated between LSCI and IRT, after a cold stimulus (*p* < 0.01), poor correlation was present between objective assessments and the Raynaud Condition Score diary (*p* > 0.05). Reproducibility of IRT and LSCI was acceptable
	Wilkinson et al., 2018	159 SSc	LSCI and thermography had a good reliability after the reperfusion/rewarming test.
	Lambrecht et al., 2016	40 SSc	LASCA demonstrated very good inter-rater reliability of PBP measured at the distal fingertips levels
	Ruaro et al., 2018a	62 SSc and 62 HS	Skin PBP was analyzed by LASCA at the level of dorsum of the middle phalanx of the third fingers, dorsal aspect of the hands and zygoma The DT was assessed by both skin high frequency ultrasound (US) and mRSS in the same above reported areas. At the level of finger dorsum a statistically significant negative correlation was observed in SSc patients between skin BP and both ultrasound-DT and mRSS, but not at the level of hand dorsum and zygoma. A progressive decrease of skin BP and increase of ultrasound-DT was found correlated with the progression of the severity of nailfold videocapillaroscopy pattern of microvascular damage (“Early,” “Active,” or “Late”)
	Ruaro et al., 2015	20 SSc	LASCA was used to monitor the perfusion of fingertip DU before and after 10 days of local/systemic treatment. Regions of interest (ROI) to analyze BP were created al the level of ulcer, peri-ulcer, periungual and fingertip areas. A statistically significant increase of BP was observed during the follow-up in the DU ROI. A significant decrease of BP was observed in the peri-ulcer area. A statistically significant decrease of ulcer size was measured by LASCA.
	Gigante et al., 2021	176 SSc and 142 HS	Median peripheral blood perfusion evaluated by LASCA was significantly lower for SSc patients than healthy controls.
	Domsic et al., 2014	15 SSc and 15 HS	LSCI revealed a distinct pattern of microcirculatory abnormalities in response to ischemia in SSc patients compared to controls.
	Trombetta et al., 2016	30 SSc	LASCA was used to assess PBP during longterm therapy with the endothelin receptor antagonist bosentan (BOSE) and the synthetic analog of prostacyclin PGI_2_ iloprost (ILO). The follow-up period was 4 years. ILO + BOSE group showed a progressive increase of PBP
	Ruaro et al., 2018b; Ruaro et al., 2019c	46 PRP, 46 SSc	LASCA was also used to assessed for 6-month the skin blood perfusion at the level of hand during aminaphtone treatment. Raynaud’s condition score (RCS) and both frequency and duration of Raynaud’s attacks were also assessed. Aminaphtone treated patients showed a progressive statistically significant increase of PBP, RCS, frequency of Raynaud’s attacks/day and their duration, in all skin areas
LASCA in systemic lupus erythematosus	Ruaro et al., 2021	20 SLE, 20 PRP and 20 HS	PBP was detected by LASCA in different regions of the hand and at the facial level. Patients with SLE and PRP had significantly lower PBP levels than those of HS in 3 hand areas (fingertip, palm, and periungual; *p* < 0.01). However, the SLE, primary RP and HS groups had comparable BP values at the hand dorsum and face.

*HS, healthy subjects; LASCA, laser speckle contrast analysis; PBP, peripheral blood perfusion; SSc, systemic sclerosis; PRP, primary Raynaud’s phenomenon; HS, healthy subjects; LSCI, laser speckle contrast imaging; IRT, infrared thermography; SLE, systemic lupus erythematosus.*

The same authors assessed the PBP of 70 SSc patients and 70 gender and age matched HS by LASCA. They evaluated different face areas, i.e., the forehead, nose tip, zygomas and perioral region and the dorsal and volar aspects of hands. Their data confirmed a significantly lower PBP in SSc patients than in HS at the fingertips, in periungual areas and the palms of the hands but not at the level of the dorsum of hands, whole face and the different facial areas (Sulli et al., 2014b; Ruaro et al., 2016). Moreover, there was a negative correlation between PBP values and loss of capillaries in all three hand areas. The SSc patients with diffuse cutaneous involvement (dcSSc) had a lower PBP at the fingertips, periungual areas and the palms of hands (*p*-value < 0.05), than those with limited cutaneous SSc (lcSSc) but not at the dorsum of the hands or the face (Sulli et al., 2014b; Ruaro et al., 2016). There was a 95% reproducibility of the LASCA assessment, which was performed by the same operator in all SSc patients and controls (Sulli et al., 2014b; Ruaro et al., 2016). The same group made a LASCA PBP evaluation of various skin areas of the hands and face in 31 primary RP patients (PRP), 70 SSc patients and 68 HS. Both the PRP and the SSc patients had a statistically significantly lower PBP than the HS at the level of the fingertips, the periungual area, the palmar aspect of the 3rd finger and in palm areas. Moreover, there was a significantly lower PBP in PRP patients than in SSc patients with the “Early” microangiopathy pattern in the same aforementioned areas (Ruaro et al., 2019a).

Jeon et al. (2020) assessed skin blood perfusion by LSCI in 10 SSc female patients with lcSSc or dcSSc who had facial and peri-oral fibrosis which limited their mouth opening and compared the data with those of 10 HS. Peri-oral and lip LSCI measurements were compared to mouth-opening measurements (measured in cm) using a plastic cone and mouth handicap index in SSc scores. There was a good correlation between peri-oral and lip LSCI to mouth-opening in SSc patients. However, no significant difference was observed between SSc and HS at the peri-oral area, in line with other previous studies (Jeon et al., 2020).

Della Rossa et al. (2013) studied blood flow by LASCA at the level of hand dorsum at baseline and after both cold test and post-occlusive reactive hyperemia (PORH) test in 20 PRP patients, 36 SSc patients and 20 HS. They reported that SSc patients had a significant reduction of blood flow after the cold test, compared to HS (*p*-value = 0.01) (Della Rossa et al., 2013). Furthermore, SSc patients required a statistically significantly longer recovery time than HS or PRP patients. The homogeneous pattern of flux distribution differed significantly in HS (95%), PRP (80%) and SSc patients (16%) (*p*-value < 0.0001). These observations evidenced a difference in the microcirculation dynamics of SSc patients compared to PRP and HS patients and may help to distinguish early versus established disease (Della Rossa et al., 2013). The latter hypothesis was further tested by the same group in 60 SSc patients which were divided into full-blown SSc (*n* = 50) and those with a very early SSc diagnosis (VEDOSS) cases (*n* = 10). They observed that the PORH decrease in the peak flow depended on the capillaroscopic pattern (*p*-value = 0.0027) and the capillary density reduction (*p*-value < 0.01). Moreover, there was a statistically significant difference in the PORH peak flow between VEDOSS and established SSc patients (*p*-value = 0.0011) evidencing a different pattern of vascular involvement in VEDOSS patients compared to those with established disease, mirroring the capillaroscopic changes (Della Rossa et al., 2016).

The study of Domsic et al. (2014) revealed a distinct pattern of microcirculatory abnormalities, evaluated by laser speckle contrast imaging (LSCI), in response to ischemia in SSc patients compared to HS. Interesting, the LSCI evaluation demonstrated a blunted microcirculatory hyperemia of the hand with greater subsequent response to nitroglycerin (Domsic et al., 2014).

Pauling et al. (2012) evaluated the dynamic assessment of digital vascular perfusion after a standardized local cold challenge by LSCI and infrared thermography (IRT) in 14 HS. They reported a good correlation between LSCI and IRT (Pauling et al., 2012). Later, the same group made a cross-sectional evaluation of 25 SSc and 18 PRP patients by LSCI, IRT and the Raynaud Condition Score (RCS) diary (Pauling et al., 2015). All the patients had a simultaneous assessment of their digital perfusion by LSCI and IRT, with two cold challenges, 2 weeks apart (*p*-value < 0.05). The reproducibility of IRT and LSCI was acceptable (ICC 0.51–0.63) and there was moderate-to-good correlation between LSCI and IRT (*p*-value < 0.01) but a poor correlation between the objective assessments and the RCS diary (*p*-value > 0.05). Neither subjective nor objective assessments differed between primary RP and SSc patients (Pauling et al., 2015).

Lambrecht et al. (2016) reported a very good inter-rater reliability of LASCA in 40 SSc patients. It was measured at the distal fingertip levels by two independent raters, under stable environmental and instrumental settings. A recent study also reported that LASCA has a very good inter-rater reliability for peripheral blood perfusion measurements in SSc (Lambrecht et al., 2016), where significantly lower PBP levels were observed in SSc patients than in HS, in various skin areas (Roustit et al., 2010; Pauling et al., 2012; Ruaro et al., 2014, 2019a; Della Rossa et al., 2016).

Wilkinson et al. (2018) carried out a multicenter study on 159 SSc patients and evaluated the validity/reliability of responses to the hand cold challenge, measured by LSCI and thermography. It was observed that LSCI and thermography had a substantial reliability after the reperfusion/rewarming test, with high convergent validity (Wilkinson et al., 2018).

In conclusion, all these studies demonstrated that laser speckle technique can help in the evaluation of functional microvascular impairment.

## Correlation With Organ Involvement

### Laser Speckle Contrast Analysis in Lung Disease

The aforementioned study by Della Rossa et al. (2013), which assessing a total of 76 subjects (20 HS, 20 PRP and 36 SSc patients) measured the cutaneous blood flow by LASCA, applied at the dorsum of the hand to evaluate qualitative parameters, such as the presence of proximal distal gradient and the homogeneity of the flux distribution. They reported no statistically significant differences in the basal values, after occlusive/ischemic and the cold test, when patients were stratified according to the type of organ involvement, i.e., interstitial lung disease (ILD) or pulmonary arterial hypertension (PAH) (*p*-value > 0.05) (Della Rossa et al., 2013).

In 2013, LASCA was used to assess PBP during long-term therapy with the endothelin receptor antagonist bosentan and the synthetic analog of prostacyclin PGI_2_ iloprost in 30 SSc patients. There was no statistically significant correlation between the PBP or both DLCO and PAH, during the study period. However, there was a significant reduction in the incidence of new DU, whereas DLCO and PAH did not worsen during follow-up (Trombetta et al., 2016).

Gigante et al. (2021) evaluated peripheral blood perfusion and the proximal-distal gradient (PDG) of the hands as biomarkers of SSc major vascular complications (digital ulcers, pulmonary arterial hypertension, scleroderma renal crisis) and mortality by LASCA. Interesting, the researchers observed that PDG predicts major vascular complication and 5-year mortality of SSc patients (Gigante et al., 2021).

However, the role of LASCA to predict organ involvement in SSc patients remains to be clarified.

### Laser Speckle Contrast Analysis in Dermal Thickness

In 2014, for the first time, a study demonstrated a relationship between nailfold microangiopathy severity by NVC, finger dermal thickness (DT) by ultrasound (usDT) and peripheral blood perfusion (PBP) by laser, in 57 SSc patients and 37 HS. A negative correlation was observed between PBP and both usDT (*p*-value = 0.007) and the modified Rodnan skin score (mRSS) values (*p*-value = 0.0002). The SSc patients had a higher usDT at the finger level and a lower FBP than HS (*p*-value < 0.0001) (Sulli et al., 2014a). Ruaro et al. (2018a) enrolled 62 SSc patients and 62 HS, evaluating them with LASCA, usDT and the mRSS at the level of dorsum of the middle phalanx of the third fingers and the dorsal aspect of the hands and zygoma. The study demonstrated a statistically significant negative correlation between skin PBP and both usDT (*p*-value = 0.0005) and mRSS (*p*-value = 0.0007) at the finger level but not at the level of the hand dorsum or zygoma. A progressive decrease in skin BP and an increase in usDT was correlated with the progression of the severity of the NVC patterns (“Early,” “Active,” or “Late”) (Ruaro et al., 2018a).

These data supported the cutaneous histopathological data, which evidenced the presence of vasculopathy as a universal feature in SSc, whatever the extent of skin fibrosis. Moreover, the skin fibrosis distribution tended to follow the cutaneous sites typically involved in thermoregulation, i.e., the fingers, forearm, face and feet. This observation was confirmed by the significantly different results observed at the digital level (affected in the majority of the patients) but not in the more proximal sites (Gaillard-Bigot et al., 2014; Ruaro et al., 2019a).

### Laser Speckle Contrast Analysis in the Follow-Up and Prediction of Digital Ulcers

Laser speckle contrast analysis has been used to monitor the perfusion of DU in 20 SSc patients with recent onset of fingertip DU, before and after 10 days of local/systemic treatment (Ruaro et al., 2015). Specific ROI were created to analyze the PBP at the level of the ulcer, peri-ulcer, periungual and fingertip areas. A statistically significant increase in PBP was observed during the follow-up in the ROI created at the level of the DU area (*p*-value < 0.0001) and in the peri-ulcer area (*p*-value < 0.0001). Moreover, LASCA evaluation evidenced a statistically significant decrease in ulcer size (*p*-value < 0.0001) (Ruaro et al., 2015). Another case report study by the same authors demonstrated that LASCA is able to make a good evaluation of the PBP variation at long-term follow-up and allows for a safe long-term monitoring of DU evolution in SSc patients (Ruaro et al., 2015). In conclusion, LASCA seems to be a safe, non-contact method to monitor DU evolution in SSc patients, by the evaluation of blood perfusion and areas involved by DU during treatment.

Barsotti et al. (2020) evaluated the role LASCA plays in predicting the outcome of 40 DU in 31 SSc patients with SSc. The LASCA analysis evidenced significantly lower average blood flow values at the fingers affected by DU (*p*-value = 0.036) and in the periulcer area (*p*-value = 0.041) of dcSSc compared to those of lcSSc patients. The presence of infection was associated to a significantly higher blood flow at the finger with DU, at the ulcer level and in the peri-ulcer area (*p*-value < 0.05) (Barsotti et al., 2020).

Summarizing, these studies demonstrated that LASCA may help in the prediction of the healing time of DU in SSc patients (Ruaro et al., 2015; Barsotti et al., 2020).

### Laser Speckle Contrast Analysis Correlates With Therapy Effects

In 2013, LASCA was used to assess PBP during long-term therapy with the endothelin receptor antagonist bosentan and the synthetic analog of prostacyclin PGI_2_ iloprost (Trombetta et al., 2016). Thirty patients with SSc already receiving intravenous Iloprost (80 μg/day) for 5 continuous days (every 3 months), were recruited into the clinic study. Fifteen patients continued treatment (the Iloprost group), whilst Bosentan was added to the treatment of the other 15 patients (125 mg twice/day) (the Combination group), administered at the onset of PAH or DU. Observations made at a 4-year follow-up sowed that the Combination group had a progressive PBP increase and absolute capillary number/mm, supporting the hypothesis that the combined therapy protocol may lead to a progressively significant recovery in both the structure and function of microvasculature, linked to improved clinical outcomes, whatever the disease severity (Trombetta et al., 2016).

In 2019, LASCA was also used to assess PBP at the level of the hand over a 6-month treatment period with aminaphtone in 46 patients with PRP or SSc-RP (Ruaro et al., 2018b,2019c). The patients treated by aminaphtone had a progressively statistically significant PBP increase and as a decrease in RCS, frequency of Raynaud’s attacks/day and their duration, in all skin areas. Five weeks after aminaphtone discontinuation, the PRP values were still significantly higher than those at baseline in most of the skin areas in both primary and secondary RP patients. This study demonstrates that aminaphtone is able to increase skin BP and improve the clinical symptoms of RP, with sustained efficacy of up to 6 months, even in patients with SSc (Ruaro et al., 2018b,2019c).

In conclusion, functional assessment of the peripheral microvasculature can help to evaluate the response to therapy in SSc patients.

## Laser Speckle Contrast Analysis in Systemic Lupus Erythematosus

Systemic lupus erythematosus is an autoimmune disease, defined by numerous clinical and serological manifestations, including vascular involvement (Cutolo et al., 2018; Tamirou et al., 2018; Fatemi et al., 2019; Ruaro et al., 2021). Few studies have evaluated morphological anomalies in the SLE microcirculation and, to the best of our knowledge, they have not assessed the correlation between the morphological and functional damage. A very recent study evaluated PBP by LASCA in various cutaneous regions of the hands and face of 60 subjects (20 SLE, 20 PRP patients and 20 HS) (Ruaro et al., 2021). SLE and PRP patients had significantly lower PBP levels than the HS in 3 hand areas (the fingertip, palm and periungual areas; *p*-value < 0.01). However, there was no significant difference in the PBP of the hand dorsum or face in any of the groups. NVC assessment evidenced a positive correlation between the number of capillaries and the PBP levels in the periungual, fingertip and palm of hands (*p*-value < 0.01), only in SLE patients (Ruaro et al., 2021). In conclusion the study demonstrated a correlation between functional and morphological microvascular impairment in SLE patients. The study concluded by recommending the importance for clinicians not to underestimate the risk of subclinical microvascular dysfunction in rheumatic diseases other than SSc, such as SLE, which are still characterized by endothelial and vascular alterations at different levels (Cutolo et al., 2018; Fatemi et al., 2019; Ruaro et al., 2021).

## Conclusion

Microvascular damage and dysfunction are the earliest morphological and functional markers of several connective tissue diseases, including SSc and SLE (Cutolo et al., 2014, 2018; Soulaidopoulos et al., 2017; Pauling et al., 2019; Perelas et al., 2020; Orlandi et al., 2021; Ruaro et al., 2021). This makes morphological and functional assessment of the peripheral microvasculature a must for the diagnosis, prognosis and evaluation of specific therapeutic effects in patients with connective tissue disorders (Cutolo et al., 2014, 2018; Soulaidopoulos et al., 2017; Pauling et al., 2019; Perelas et al., 2020; Orlandi et al., 2021; Ruaro et al., 2021). Indeed, laser speckle technique is able to detect significant reductions in PBP in connective tissue diseases patients, where PBP is specifically impaired in those areas typically affected by Raynaud’s phenomenon, i.e., the fingertips, periungual and palm areas, with early complications such as DU (Ruaro et al., 2015; Barsotti et al., 2020), correlated to a more advanced microvascular anatomical damage (Ruaro et al., 2014).

Lastly, laser speckle techniques may safely monitor DU evolution in SSc patients during standard treatment. In SLE patients the use of these techniques may help clinicians not to underestimate the risk of subclinical microvascular dysfunction (Tamirou et al., 2018). Furthermore, several studies have reported on the good reliability of laser speckle techniques (Roustit et al., 2010; Lambrecht et al., 2016).

This study emphasizes the utility of laser speckle techniques in providing a comprehensive view of the spatial heterogeneity of microvascular dysfunction and the fact that they may well add significant functional information to the morphological picture offered by NVC. Furthermore, ischemic challenge and cold test allow for the study of regional differences in skin perfusion that may help to distinguish between primary and secondary RP (Della Rossa et al., 2013, 2016; Ruaro et al., 2019a). Indeed, these techniques also have a prognostic significance and may help in the *in vivo* evaluation of the real time effect of pharmacological challenge on selected areas so as to avoid the potentially harmful administration of vasodilator treatment (Trombetta et al., 2016; Ruaro et al., 2018b,2019c).

However, further studies on larger cohorts would provide further much needed data as to correlations between blood perfusion assessed by laser speckle techniques and internal organs, to validate and support the use of these techniques in daily practice and clinical trials.

## Author Contributions

BR, CB, MC, and FS made substantial contributions to the conception and design of the work, drafted the manuscript and revised it critically for important intellectual content and the final approval of the version to be published. BW, EB, PC, CA, PG, and MB drafted the manuscript and revised it critically for important intellectual content and the final approval of the version to be published. All authors contributed to the article and approved the submitted version.

## Conflict of Interest

The authors declare that the research was conducted in the absence of any commercial or financial relationships that could be construed as a potential conflict of interest.

## Publisher’s Note

All claims expressed in this article are solely those of the authors and do not necessarily represent those of their affiliated organizations, or those of the publisher, the editors and the reviewers. Any product that may be evaluated in this article, or claim that may be made by its manufacturer, is not guaranteed or endorsed by the publisher.
